# Lessons from COVID-19: Perspectives of senior managers and decision-makers in substance use services in Wales

**DOI:** 10.1186/s13011-026-00714-9

**Published:** 2026-03-12

**Authors:** Shannon Murray, Katy Holloway, Marian Buhociu, Martin Blakebrough, Caroline Phipps

**Affiliations:** 1 University of South Wales, Llantwit Rd, Treforest, Pontypridd, CF37 1DL UK; 2 Kaleidoscope, Cardiff, UK; 3 Barod, Newport, UK

**Keywords:** COVID-19, Substance use services, Inequity, Third sector, Digital divide, Wales

## Abstract

**Background:**

The COVID-19 pandemic significantly disrupted substance use services, potentially exacerbating existing inequities in access to treatment. This study examines the perspectives of senior managers and decision-makers in Wales regarding equity issues in substance use services during the pandemic.

**Methods:**

Semi-structured interviews were conducted with fifteen senior managers and decision-makers from various organisations in the substance use field in Wales. Interviews were conducted during autumn 2020. Data were analysed using reflexive thematic analysis to identify key themes related to equity in access to treatment.

**Results:**

Key findings highlighted significant disparities between third sector and statutory NHS services, particularly in access to PPE, and recognition as essential workers. The rapid shift to digital service provision exposed a digital divide, potentially excluding vulnerable service users. However, the pandemic also accelerated innovations in service delivery, such as the introduction of long-acting injectable buprenorphine and revised take-home medication policies. Long-term concerns included the economic impact on vulnerable populations, potential increases in mental health issues and substance use, and funding uncertainties for third sector services.

**Conclusions:**

The pandemic exposed and exacerbated existing inequities in substance use services while also driving rapid innovation. Findings suggest a need for greater recognition and support for third sector services, strategies to address the digital divide, and sustained funding to ensure equitable access to treatment. These insights can inform policy and practice to develop more equitable and resilient substance use services in preparation for any future crises.

## Background

The COVID-19 pandemic has had a profound impact on healthcare services worldwide, with substance use services facing unique challenges in maintaining support for vulnerable populations [1]. To fully understand these challenges and their implications, it is important to examine the concept of inequity in healthcare access, particularly in the context of substance use services. Inequity in healthcare refers to systematic disparities in access, quality, or outcomes of health services that are unnecessary, avoidable, and unjust. In substance use services, inequity manifests as unequal access to treatment and support, often exacerbated by societal factors such as stigma, resource constraints, geography, and systemic biases [2–4].

Prior to the pandemic, various groups experienced significant inequities in accessing substance use services, including women facing childcare-related barriers [5–7], rural populations facing geographical isolation [8, 9], LGBTQ+ individuals encountering discriminatory practices [10], minoritised ethnic groups facing cultural and linguistic barriers [11–14], and those from lower socioeconomic backgrounds struggling with financial constraints [15]. These pre-existing inequities provide important context for understanding how the pandemic affected access to services.

In Wales, substance use services are delivered through a mixed economy of care, with commissioning coordinated through seven regional Area Planning Boards (APBs) using Welsh Government funding [16] The third sector, comprising charitable organisations such as Barod, Kaleidoscope, and others, delivers a substantial proportion of community-based treatment, psychosocial interventions, and harm reduction services. NHS services primarily provide clinical prescribing through community drug and alcohol teams, hospital-based interventions, and some specialist treatment [17]. Public Health Wales, as the national public health agency, provides strategic guidance, surveillance data, and public health coordination [18]. Criminal Justice partners, including probation services, work with people who offend who have substance use issues through mechanisms such as Drug Rehabilitation Requirements (DRRs) and Alcohol Treatment Requirements [19]. This service landscape is important context for understanding the findings presented in this paper.

The onset of the COVID-19 pandemic and subsequent lockdown measures forced rapid adaptations in substance use services, often exacerbating existing inequities in access to treatment [20–22]. Services across the UK, including Wales, faced unprecedented challenges in maintaining support for vulnerable populations while adhering to public health restrictions [23, 24]. In response, substance use services implemented various strategies to maintain continuity of care [24], including shifting to online and telehealth services, distributing low-cost smartphones to maintain connectivity with service users, and implementing take-home medication policies for opioid substitution therapy[21, 25, 26]. While these adaptations helped maintain some level of service provision, they also introduced new challenges and potentially widened existing inequities [24].

As the pandemic unfolded, a digital divide emerged, limiting access for individuals without reliable internet or digital literacy [4]. Disruptions to harm reduction services created additional challenges in maintaining needle exchange programmes and supervised consumption sites [27]. These impacts varied considerably across demographic groups, with particularly pronounced effects based on socioeconomic status, ethnicity, and geographical location [28].

### Current study

While recent research has documented service users’ experiences of these changes [29], there remains a critical gap in our understanding; the perspectives of senior managers and decision-makers who orchestrated these service adaptations. Their insights are particularly valuable as they possess unique knowledge of system-level challenges and were responsible for directing the implementation of rapid changes in service delivery during this unprecedented crisis.

This study, embedded within the broader ‘Lessons from COVID-19 for substance use services’ project in Wales [24], aimed to address this knowledge gap by examining how senior figures perceived and responded to equity issues during the pandemic.

This paper focuses specifically on three dimensions of inequity that emerged from the data: (1) inequity between third sector and statutory NHS services in terms of resources, recognition and support; (2) inequity in access to services arising from digital exclusion; and (3) the broader inequity faced by substance use services and their clients compared to other healthcare areas, reflecting persistent stigma.

Our primary objectives are: to explore senior managers’ and decision-makers’ perspectives on how the COVID-19 pandemic impacted equity in access to substance use services in Wales; to identify the key equity challenges faced by substance use services during the pandemic and how these were navigated; to examine the adaptations and innovations implemented to ensure equitable access to services and their perceived effectiveness; and to understand the long-term concerns and priorities for maintaining and improving equity in substance use services in the post-pandemic context.

The insights gained from this research have particular relevance for current policy development and practice improvement. As the UK COVID-19 inquiry [30] progresses, our findings can directly inform future pandemic preparedness efforts by ensuring that equity considerations in substance use services are prominently featured in policy discussions.

## Methods

Qualitative semi-structured interviews were conducted with fifteen senior managers and decision-makers working in (or alongside) substance use services in Wales. Participants were sampled to represent a range of organisations, including third sector treatment agencies, Welsh Government, Public Health Wales, Area Planning Boards, and Criminal Justice agencies. Purposive sampling was employed to ensure that the study captured the perspectives of individuals in key strategic and decision-making positions within the substance use sector in Wales. This approach allowed for the selection of participants who could provide rich, in-depth insights at a policy and practice level.

### Data collection and analysis

Interviews were conducted by a single researcher (KH) via telephone or online (Teams or Zoom) during autumn 2020. The use of a single interviewer ensured consistency in the data collection process and allowed for the exploration of emerging themes across interviews. The average interview duration was 76 min, enabling participants to provide detailed accounts of their experiences and perspectives.

The interviews followed a semi-structured format, with a pre-determined set of questions used to guide the conversation while allowing flexibility for participants to raise additional relevant issues. The interview guide covered topics such as the impact of the pandemic on substance use services, equity challenges encountered, adaptations and innovations implemented, collaboration and communication, and long-term concerns and priorities.

All interviews were digitally recorded with participants’ consent and transcribed verbatim by a professional transcription service. The transcripts were then analysed using NVivo, a qualitative data analysis software. A reflexive thematic analysis approach was employed, involving an iterative process of coding, reviewing, and refining themes [31–33]. The coding framework was developed inductively by the primary researcher (KH). Initial codes were generated through a close reading of the transcripts, which were then organised into broader themes and sub-themes. The emerging themes were discussed with the wider research team, which included an experienced qualitative researcher as well as experts by experience and profession. This collaborative process helped to ensure the credibility and trustworthiness of the findings.

To protect the identity of our interviewees (given that our sample consisted of an ‘elite’ group, making them more easily identifiable) we have opted against including interviewee ID numbers after each quotation to prevent readers from linking quotations and potentially identifying the people who said them [34].

### Ethical considerations

Ethical approval for the study was granted by the University of South Wales Faculty of Life Sciences and Education Ethics Committee (Reference: 20803LR). All participants provided informed consent prior to the interviews, and the study adhered to the core ethical principles of avoiding harm and deception while maintaining confidentiality and providing anonymity.

### Sample characteristics

The study sample consisted of fifteen participants, with a relatively balanced gender distribution of eight males and seven females. Participants were drawn from various organisation types, with the largest group (*n* = 6) coming from third sector treatment agencies. The remaining participants were evenly distributed across Welsh Government (*n* = 2), Public Health Wales (*n* = 2), Area Planning Boards (*n* = 3), and Criminal Justice (*n* = 2). Criminal Justice participants included representatives from probation services and organisations working with offenders who have substance use issues; during the pandemic, their role included maintaining contact with people under supervision, adapting supervision requirements, and coordinating with treatment services. In terms of experience in their current senior roles, participants had an average of nearly 10 years, with experience ranging from a minimum of two years to more than twenty years. The sample was fairly evenly split between those with 10 + years of experience (*n* = 8) and those with less extensive experience (*n* = 7).

It is worth noting that direct NHS service provider perspectives are not represented in this sample. While their inclusion would have provided valuable additional insights, the intense pressures on NHS services during the peak of the pandemic made it unfeasible to secure approval for their participation within the study period. However, this limitation is partially mitigated by the strong representation of third sector organisations, which played a crucial frontline role throughout the pandemic, and the inclusion of participants from Public Health Wales, Welsh Government, and Area Planning Boards who interface with NHS services. Indeed, the substantial third sector participation in this study reflects their significant contribution to substance use service provision in Wales. Their perspectives offer particularly valuable insights into the challenges and innovations in service delivery during this unprecedented period.

## Results

The analysis of the interview data revealed five key themes related to equity in access to substance use services during the COVID-19 pandemic. These themes are presented below, illustrated with representative quotations from participants.

### Theme 1

Disparities in Service Provision During the COVID-19 Pandemic.

A central theme that emerged from the interviews was the perceived disparity in how various parts of the healthcare system were resourced and prioritised during the pandemic, with particular concerns raised around third sector substance use services.

### Access to PPE

Interviewees expressed frustration about the lack of access to PPE for third sector service staff. One interviewee described how early in the pandemic his role was *‘very much about begging*,* borrowing stealing*,* finding*,* sourcing any kind of PPE.’* While PPE shortages affected many healthcare services in the early pandemic, interviewees felt that third sector substance use services faced particular barriers in accessing these essential resources.

The inequity of access to PPE was starkly illustrated by another interviewee:

*The one thing that I still stand by*,* that was massively let down is… we had no access to PPE. That is something that [organisation] and our partners had to source for ourselves from day one*,* had to get. We kept hearing all of the news that PPE was being made available*,* but that just was not the case*

One interviewee frustrated by the inequity in access to equipment that would help to protect them strongly felt that “*Public Health should be* ***public*** *health*,* not just NHS help.”*

This experience highlighted broader questions about how various parts of the healthcare system are valued and resourced during emergencies. Several participants noted this as an important lesson for future pandemic planning, with one explaining that there needs to be a *‘common understanding right from the start about what their contribution is and what recognition they require and they’ve provided the same PPE*,* access to testing*,* statuses of their colleagues in statutory services.’*

The tangible impact of these inequities was described by one third sector manager who detailed their struggle to access basic protective equipment:

*We weren’t being treated equitably*,* so we weren’t being treated like a statutory service. So*,* the statutory health services were provided with protective equipment*,* and I’m talking masks*,* hand gel*,* gloves*,* in some cases aprons*,* and we couldn’t access those basic pieces of equipment*,* and so we had an ongoing battle with various strategic people about getting hold of that equipment or getting supplies of that equipment ourselves*

### Recognition as essential care workers

This perceived disparity in access to resources was compounded by a perceived lack of recognition for third sector staff as essential workers. Participants expressed frustration and disappointment at the exclusion of third sector substance use workers from the £500 bonus payment awarded to other health and social care staff during the pandemic. This was seen as a devaluation of the vital contributions made by the third sector in supporting vulnerable populations.

*One of the things that we were really disappointed about was*,* our workers are considered care workers*,* and yet they weren’t given the £500 bonus that all the other care workers were given … so that made us very angry*

This disparity in recognition extended beyond financial rewards to a general lack of acknowledgment of the third sector’s crucial role:

*I think as well*,* the whole understanding of the workforce in the third sector about what* [they do] *… they are the equivalent of social workers and health workers and people need to recognise that*

### Organisational responsiveness and flexibility

The disparity between the treatment of the third and statutory sectors was at odds with the excellent work of the third sector, which was *“staying open”* and providing a critical *“front door”* to connect with people in need during the lockdown period. Several interviewees commented on how the third sector was able to respond quickly (like a *“nippy little motorboat”)* to the demands of the pandemic and praised its agility, flexibility, and responsiveness at a time of crisis.

The disparities exposed by the pandemic were seen as a reflection of deeper, systemic inequities in the way substance use services are funded, commissioned, and delivered. Interviewees called for a fundamental shift in how the third sector is valued and supported, arguing that the pandemic should serve as a catalyst for greater collaboration, resource sharing, and mutual respect between statutory and third sector services.

Third sector services were described as ‘nimble,’ ‘flexible,’ ‘responsible’ and ‘reactive,’ while statutory services were characterised as slower to adapt due to more rigid decision-making hierarchies. One participant explained, statutory services were like *‘tankers’* that were difficult to steer in a different direction, particularly during crisis situations where quick responses were needed. This was attributed to their organisational structure, with another interviewee noting that statutory services *‘have to wait for directives*,* and their directive is often right up the chain.’*

*I think [the statutory] health [sector] really struggled. The statutory agencies I thought were very poor and slow*,* particularly Criminal Justice*

Despite these challenges, the pandemic demonstrated the potential for rapid service transformation when necessary. One interviewee described how the work in getting services up and running and delivering online support was ‘*very much like five years of work done in a week.’*

Another interviewee called for people to stand up for third sector services, which in their view, had been *‘relegated’* and *‘disparaged.’* This interviewee urged for more assertiveness in supporting and lobbying for third sector workers. Similarly, another interviewee reflected on the need to stand up for people with substance misuse problems.

*I wonder whether we should have pushed more … I mean I don’t know whether that would’ve got me anywhere. I think maybe with hindsight you look back and this thing of almost rolling over and playing dead by saying*,* ‘Yeah*,* okay guys*,* your guys are more important than mine’ was*,* with hindsight looking back on it*,* was a load of bollocks because actually … maybe we would’ve got them in earlier and obviously there’s accumulated extra healthcare issues in the meantime*

#### Theme 2

Digital Divide in Service Access.

Another important theme that emerged from the interviews was the impact of the rapid shift to digital service delivery on equity in access to support. Participants recognised that while digital platforms offered certain advantages, such as increased flexibility and reduced travel time for staff, they also risked creating new barriers for service users who were already facing significant challenges. Concerns were raised about the ability of service users who were homeless, lacked stable housing, or had limited financial resources to access the necessary devices and internet connectivity to engage with remote support.

*People were self-isolating left*,* right and centre. We were trying to move things to digital inclusion*,* but that was very difficult for a lot of our service users*,* because they didn’t have the kit. They didn’t have the IT stuff*

In addition to issues of access to technology, participants also highlighted the challenges posed by the digital literacy gap. Many service users, particularly those from older age groups or with complex needs, were seen as lacking the skills and confidence to navigate online platforms and engage effectively with remote support. This raised concerns about the potential for the shift to digital delivery to reinforce existing inequities and leave vulnerable service users behind.

*The other issue that we had was*,* a lot of our people aren’t digitally included. They don’t have those digital skills. They might have a phone*,* but they might not be able to work Teams or Zoom or… So*,* we knew there was a training issue there*,* and confidence; confidence to use those digital platforms*

To mitigate the impact of the digital divide, interviewees described various strategies their services had employed, such as providing devices and data packages to service users, offering digital skills training and support, and maintaining COVID-safe face-to-face provision for those unable to engage remotely. However, there was a recognition that these efforts were often piecemeal, and that a more systematic, well-resourced approach was needed to truly bridge the digital divide.

*It’s about choice isn’t it*,* it’s about being able to provide a whole range of interventions*,* so it doesn’t have to be just one way or the other*,* we can do lots of different things and we just need to make sure that people have got equal access to those services*,* I think that’s the important thing*

Interviewees emphasised the need for a blended approach to service delivery, combining remote and face-to-face support to ensure that no one was excluded. There was recognition that while digital platforms could play a key role in enhancing access for some, they should not be seen as a panacea or a replacement for in-person care.

#### Theme 3

Stigma and Recognition

The fact that third sector substance use services were not recognised as ‘essential’ was also seen by many participants as an extension of the stigma experienced by people who use drugs and alcohol. The exclusion of third sector workers from ‘key worker’ status and the £500 bonus payment was also felt to reflect a broader societal devaluation of the lives and needs of those struggling with substance use.

*I think the stigma that people were seeing*,* our workforce feeling that the stigma that’s attached to working in drugs and alcohol services*,* that somehow*,* it’s kind of not as important as other services*

*The £500 for key workers*,* that was a bit of a kick in the teeth for our staff*,* you know*,* it’s kind of like ‘well you’re not as important as NHS staff’ and I think that’s something that we’ve always battled against*,* that kind of stigma and discrimination*

Participants argued that this lack of recognition was rooted in the persistent stigmatisation of substance use as a moral failing rather than a health issue. They described how this stigma permeated not only public attitudes but also decision-making at a policy and funding level, with substance use services often seen as a low priority compared to other areas of health and social care.

*There’s still a lot of stigma*,* there’s still a lot of people who think ‘well*,* it’s their own fault*,* they’ve chosen to take drugs so why should we help them?’ and I think that that’s something that we’ve got to continue to challenge*

The pandemic was seen as exposing and amplifying these underlying inequities, with participants describing how the needs of people who use drugs and the services that support them were often overlooked in the crisis response. They spoke of having to fight for recognition and resources, while fearing that the impact of the pandemic on substance use and related harms was being ignored.

*I think there was a real sense of*,* you know*,* ‘where do we fit in all of this?’ because there was a lot of focus on the acute hospital sector and rightly so*,* but it felt like substance use was kind of forgotten about in the midst of all of that.*

*People that were in the throes of addiction and problematic substance use*,* where were they on the radar? Where were they on people’s agendas? It just felt like we were constantly having to remind people that we were here and that we needed to be part of the conversation.*

Participants emphasised the need for concerted efforts to challenge stigma and raise awareness of the essential, life-saving work conducted by substance use services. They called for greater recognition of the expertise and commitment of the workforce, and for substance use to be given equal priority to other areas of health and social care in policy, funding, and public discourse.

*I think we need to be much better at celebrating the work that we do and the successes that we have*,* because I think sometimes we’re not very good at that in the substance use field*,* we kind of hide our light under a bushel a bit*,* and I think we need to be much more proactive in terms of getting the message out there about the importance of the work that we do*

*We need to be a bit more assertive in terms of our lobbying and our influencing*,* and I think the pandemic has shown us that we need to be better at making the case for investment in substance use services and in challenging some of those stigmatising attitudes that still exist*

Tackling stigma and discrimination was seen as essential not only for ensuring equitable access to services but also for creating a more supportive and inclusive society for people who use drugs and alcohol. Interviewees spoke of the need for a fundamental shift in how substance use is framed and understood, moving away from a criminal justice approach towards a public health and human rights-based perspective.

*I think we’ve got to get better at educating people about the fact that substance use is a health issue*,* it’s not a moral issue*,* it’s not a criminal justice issue*,* it’s a health issue*,* and people deserve help and support just like they would if they had any other kind of health problem*

#### Theme 4

Adaptations to Ensure Equitable Access

While the pandemic posed significant challenges for substance use services, it also catalysed a wave of innovation and adaptation as services sought to maintain support for service users in the face of lockdown restrictions. Participants described a range of creative approaches that were implemented to ensure equitable access to treatment and support, while adhering to social distancing guidelines.

One key area of innovation was the rapid introduction of new pharmacological interventions, such as long-acting buprenorphine injections (Buvidal), which allowed service users to receive their medication weekly or monthly without the need for daily attendance at services. This was seen as a ‘game-changer’ in terms of reducing barriers to treatment and minimising the risk of COVID-19 transmission and according to our participants, third sector organisations had a significant role in the roll-out of this treatment.

*The pandemic has speeded up the rollout of Buvidal across Wales*,* which is fantastic because it’s removed that need for daily supervision*,* and it’s given people a lot more flexibility and autonomy in their treatment*

*In fairness to Welsh Government*,* the substance misuse team*,* with us*,* they made courageous decisions that it [Buvidal] could be a major asset across Wales*,* and we’re the only home nation to do that*

These reflections highlight how the pandemic prompted a reassessment of risk, with the risks of inaction being weighed against the risks of new interventions, ultimately driving the accelerated adoption of Buvidal.

However, some participants raised concerns about the potential unintended consequences of the rapid shift to long-acting medications, such as the risk of reduced contact with service users and missed opportunities for psychosocial support. They emphasised the need for a balanced approach that combined pharmacological interventions with ongoing support and monitoring.

*It’s great that we’ve got these new medications that can give people more freedom and flexibility*,* but we need to make sure that we’re still providing that wraparound support and that we’re not just leaving people to get on with it on their own*

Another area of adaptation was the relaxation of restrictions around take-home medications, such as methadone and sublingual buprenorphine, which allowed service users to receive larger take-home supplies and reduce the frequency of pharmacy visits. This was seen as a pragmatic response to the pandemic that had the potential to reduce barriers to treatment engagement and retention.

*The biggest thing is that we should remind ourselves that we can trust our service users to look after themselves more than we think we do. The issue is that when it goes wrong*,* it goes horribly wrong. So*,* if a service user overdoses and they die*,* they’re dead*,* and there’s no going back from that. But the reality is that across Wales*,* hundreds and hundreds and hundreds of service users were given weekly or twice-weekly pickup. We didn’t suddenly have the entire population high as a kite or dying of methadone that they bought from a mate.*

Participants reflected on how the pandemic had challenged long-held assumptions about the risks of diversion of take-home medications and forced services to confront the paternalistic and risk-averse approaches that had often characterised OST. There was a recognition that the changes prompted by COVID-19 could lead to a fundamental shift in prescribing practices and a move towards more flexible, individualised treatment options.

*I think the pandemic has really shone a light on some of the more punitive and controlling aspects of opiate substitution therapy and forced us to question whether we’ve got the balance right between managing risk and promoting autonomy*

*I would like to think that we can take some of the learning from this period and use it to inform a more person-centred approach to prescribing*,* one that recognises that people’s needs and circumstances are different and that there’s no one-size-fits-all approach*

Alongside these changes to prescribing practices, participants also described a range of other adaptations that were implemented to ensure equitable access to psychosocial support and harm reduction services. These included the provision of outreach services, the delivery of naloxone and safe injecting equipment through the post, and the use of mobile vans and outdoor settings for needle exchange and other harm reduction interventions.

*We quickly realised that we needed to take the service to the people*,* rather than expecting them to come to us*,* so we started doing a lot more outreach work*,* going out to the homeless encampments and the areas where we knew people were congregating and making sure that they had access to clean equipment and naloxone*

*We set up a postal delivery service for naloxone and injecting equipment*,* so that people could still access those vital harm reduction services even if they couldn’t come into the service in person*

While these adaptations were widely seen as positive developments that had the potential to improve access and engagement, interviewees also acknowledged that they were not without their challenges and limitations. Some expressed concerns about the sustainability of these changes in the longer term, particularly in the context of funding constraints and competing priorities.

*It’s been great to see all of this innovation and creativity in the way that we deliver services*,* but I do worry about how sustainable it is in the longer term*,* particularly if we start to see funding cuts or a shift in priorities away from substance use*

There were also concerns about the potential for some of these adaptations to exacerbate inequities in access, particularly for those service users who faced the greatest barriers to engagement. For example, the shift to postal delivery of harm reduction equipment was seen as a valuable innovation, but one that risked excluding those who were homeless or had no fixed address.

*We need to be mindful that some of these changes that we’ve made*,* while they’ve been really positive for some people*,* might actually make it harder for others to access services*,* particularly those who are most marginalised and vulnerable*

Participants emphasised the need for ongoing evaluation and monitoring of these adaptations to ensure that they were meeting the needs of all service users and not inadvertently creating new barriers to access. They also highlighted the importance of involving service users in the design and delivery of these changes to ensure that they were responsive to their needs and preferences.

*We need to make sure that we’re constantly reviewing and assessing the impact of these changes and that we’re involving service users in those conversations*,* because they’re the experts in their own lives and they know best what works for them*

The adaptations and innovations prompted by the pandemic were seen as offering valuable learning opportunities for the future of substance use services. Participants expressed hope that some of the positive changes that had been implemented, such as more flexible prescribing and a greater focus on outreach and low-threshold services, could be sustained and built upon in the post-pandemic period.

*I think the pandemic has shown us that we can be much more flexible and creative in the way that we deliver services and that we can make changes quickly when we need to. I hope that we can take some of that learning forward and use it to create a more responsive and equitable system of care*

At the same time, there was a recognition that achieving lasting change would require a concerted effort to address the structural and systemic barriers that had long hindered access to substance use services, such as poverty, homelessness, and criminalisation. Participants emphasised the need for a whole-system approach that recognised the complex and intersecting needs of people who use drugs and alcohol and that prioritised equity and social justice.

*We can’t just focus on the substance use in isolation*,* we need to look at the whole picture and address the underlying issues that are driving problematic drug and alcohol use*,* like poverty*,* trauma*,* and marginalisation. That’s the only way we’re going to create a truly equitable system of care*

#### Theme 5

Long-term Concerns for Equity

While the immediate focus of substance use services during the pandemic was on adapting to the challenges posed by lockdown and ensuring continuity of care, participants also expressed significant concerns about the longer-term impacts of COVID-19 on equity in access to services. These concerns centred around four key areas: the economic fallout of the pandemic and its impact on the most vulnerable; the potential for an upsurge in mental health problems and substance use; the sustainability of funding for services; and the wellbeing and retention of the workforce.

Participants repeatedly voiced fears about the economic consequences of the pandemic and the disproportionate impact that this would have on the most deprived communities. They noted that many of the people they supported were already living in poverty and that the loss of employment and income resulting from lockdown would only exacerbate these hardships.

*We all know there is going to be quite a big economic shock and obviously for people working in substance misuse*,* we know the impact that might have on people’s substance misuse*,* and sadly*,* Wales has got some very poor communities already who will probably take some of the biggest economic kicks*

*A lot of our clients were just about managing before the pandemic hit*,* and now they’ve lost their jobs or had their hours cut and they’re really struggling to make ends meet. We’re seeing a lot more people in crisis*,* a lot more people who are facing homelessness or food poverty.*

At the time of the interviews in autumn 2020, participants expressed significant concerns about the potential longer-term impacts of the pandemic. There were widespread worries that the economic consequences would lead to increased problematic substance use, as people turned to drugs and alcohol to cope with job losses, housing insecurity, and social isolation. As one participant explained:

*We know that there’s a strong link between poverty*,* deprivation*,* and problematic substance use*,* so we’re really worried about the impact that the economic downturn is going to have on the communities we serve. We’re already seeing an increase in alcohol-related admissions to hospital and we’re expecting to see a lot more people coming forward for treatment in the future.*

Mental health was another key area of concern, with participants anticipating that COVID-19 would both exacerbate existing mental health problems and create new ones. These concerns were rooted in their direct experience of supporting clients during the early stages of the pandemic:

*A lot of our clients have underlying mental health problems*,* like depression*,* anxiety*,* PTSD*,* and the pandemic has just made those issues so much worse…*.

*I think the mental health impact of the pandemic is going to be huge and we’re only just starting to see the tip of the iceberg…*.

Participants also expressed concerns about a potential retreat from the collaborative and risk-taking approaches that had emerged during the crisis. Some described a “cooling down” of joint initiatives and a return to risk aversion, which threatened to undermine progress made in expanding access to innovative services. This trend was seen as a concerning development that could perpetuate inequities in access to services and hinder efforts to build a more responsive and equitable system of care.

Finally, participants expressed significant concerns about the wellbeing and retention of the substance use workforce in the post-pandemic period. These concerns were particularly pressing given the sector’s pre-existing challenges with recruitment and staff retention. As one participant explained:

*Our staff have been absolutely brilliant during the pandemic*,* they’ve gone above and beyond to support service users and to keep services running. But they’re exhausted and they’re burnt out and I’m really worried about the long-term impact on their mental health and wellbeing.*

## Discussion

The COVID-19 pandemic has exposed and exacerbated long-standing inequities in access to substance use services ([1, 3], while also catalysing innovation and adaptation in the face of unprecedented challenges. The findings of this study, based on the perspectives of senior managers and decision-makers in Wales, offer valuable insights into the complex and multi-faceted nature of these inequity issues and the urgent need for action to address them [4].

A central theme that emerged from the interviews was a disparity between third sector and statutory services in terms of access to resources, recognition, and support during the pandemic [24]. Participants described how third sector services, which play a vital role in providing care to some of the most vulnerable and marginalised populations, initially struggled to access essential PPE, while these resources were more readily available to statutory health services. This lack of parity in resource allocation reflected a broader devaluation of the third sector’s essential work and, by extension, the lives of people who use drugs. This finding aligns with broader literature on the inverse care law [35], whereby those with the greatest need often receive the least resources.

The study identified significant adaptions in service delivery models during the pandemic [21]. These innovations included the rapid rollout of long-acting buprenorphine, revised take-home medication policies, and the implementation of digital services. While these adaptations helped maintain service continuity, they also revealed the sector’s capacity for rapid transformation when necessary. The successful implementation of these changes’ challenges traditional assumptions about service delivery and suggests possibilities for more flexible, person-centred approaches in the future, aligning with current UK clinical guidance which emphasises collaborative, person-centred treatment and recovery care planning [36].

The rapid shift to digital service delivery created both opportunities and challenges for equity in access [27–29]. While digital platforms increased accessibility for some service users, they risked excluding others, particularly those lacking digital resources or skills (NHS England, [4]). This digital divide highlighted the need for a balanced approach that combines technological innovation with traditional service delivery methods to ensure comprehensive access.

Participants expressed significant concerns about the pandemic’s long-term economic impact on vulnerable communities [9, 37]). The potential increase in poverty, homelessness, and social exclusion [38] threatens to exacerbate existing inequities in service access. These social determinants of health require a comprehensive response that extends beyond substance use services to address broader societal inequalities.

The findings emphasise the crucial role of service user involvement in developing equitable services [12, 13]. This aligns with Welsh Government’s commitment to co-production, as outlined in their ‘A Healthier Wales’ plan (Welsh Government, [39]). Moving away from paternalistic approaches toward collaborative service design represents a fundamental shift in how services are conceptualised and delivered. The study underscores the importance of strategic leadership in driving equity improvements [2]. Senior managers’ experiences highlight the need for clear communication, cross-sector collaboration, and flexible decision-making processes. Building system resilience requires sustained investment in workforce development, infrastructure, and innovative service models [3].

This study makes a distinct contribution to the literature on pandemic responses in substance use services. While previous research has documented service user experiences [29] and frontline staff perspectives [21], this study uniquely captures the strategic and system-level view of senior managers who were responsible for directing rapid service changes. Their insights into inter-sectoral disparities, policy barriers, and decision-making processes during crisis provide a perspective that is essential for informing future pandemic preparedness and policy development. The explicit equity lens applied to these perspectives, within the specific context of Wales’s devolved healthcare system with its strong third sector presence, offers insights that extend beyond the immediate findings to questions of how healthcare systems value and support different components of the care landscape.

### Reflections on Progress Since 2020

While this study captured perspectives from autumn 2020, it is important to consider what has transpired in the intervening years. Participants called for ongoing evaluation and monitoring of the adaptations implemented during the pandemic. Evidence of formal, systematic evaluation of equity impacts remains limited, representing a gap for future research. However, some adaptations have clearly been sustained: Buvidal continues to be available across Wales, and more flexible approaches to take-home prescribing have persisted in many areas, suggesting perceived effectiveness among clinicians and commissioners [40].

Regarding the four areas of long-term concern raised by participants, the picture is mixed. Economic pressures on vulnerable populations have continued, with cost-of-living increases creating additional hardship for many service users. Mental health presentations have increased, as participants predicted. The sustainability of third sector services faces ongoing challenges, including increased National Insurance contributions which have created significant financial pressures on charitable organisations, with the sector-wide impact estimated at £1.4bn annually [41, 42]. These financial strains risk undermining service stability. However, positive developments have also occurred, including Welsh Government’s continued commitment to integrated care and co-production as outlined in ‘A Healthier Wales’ [39]. Workforce wellbeing remains an ongoing concern, with recruitment and retention challenges continuing to affect the sector.

It is important to acknowledge that while this study’s data were collected in 2020, the ongoing relevance of these findings is shaped by subsequent policy decisions, both those that may exacerbate inequities (such as increased operational costs for third sector services) and those that may address them (such as continued investment in innovative treatment approaches). The concerns raised by participants in 2020 remain pertinent, even as the specific context has evolved.

## Limitations

The findings of this study should be interpreted in light of several limitations. Firstly, the sample size was necessarily small due to the senior status of the interviewees, which restricted the number of potential participants. The study also focused on a specific geographic context (Wales), which may limit the generalisability of the findings to other settings. Additionally, while the sample included participants from various organisations, the largest proportion (*n* = 6) came from third sector treatment agencies, with smaller numbers from statutory services. This uneven distribution may have influenced the findings; future research could benefit from a more balanced representation across sectors.

Direct NHS service provider perspectives are notably absent from this sample. This limitation means that our understanding of statutory sector decision-making processes during the pandemic, and NHS managers’ perspectives on inter-sectoral relationships and resource allocation, is incomplete. NHS managers may have had different views on the challenges and priorities during this period. The intense pressures on NHS services during data collection (autumn 2020) made their participation unfeasible, but future research should seek to include these perspectives to provide a complete and more balanced picture. This limitation is partially offset by the inclusion of participants from Public Health Wales, Welsh Government, and Area Planning Boards who interface with NHS services.

Secondly, the study relied on self-report data from a single time point, which may be subject to recall bias and may not capture the full complexity and dynamism of the issues explored. Finally, the study did not include the perspectives of frontline staff or service users, which may have provided additional insights into the equity challenges experienced during the pandemic.

## Conclusion

Despite these limitations, the study makes a valuable contribution to the evidence base on equity in access to substance use services during the COVID-19 pandemic. The findings highlight the urgent need for action to address the underlying inequities and structural barriers that have been exposed and exacerbated by the crisis, and to build a more resilient, responsive, and equitable system of care for the future. They also underscore the importance of learning from the adaptations and innovations that have emerged during the pandemic, and of harnessing the expertise and commitment of the substance use workforce to drive positive change.

As the UK continues to navigate the challenges of the pandemic and looks towards economic recovery, it is important that the lessons learned from this period are not lost but are used to inform the development of more equitable, person-centred, and sustainable models of care. This will require a concerted effort from policy makers, commissioners, service providers, and service users to work together to address the complex and intersecting challenges facing the sector, and to create a shared vision for the future of substance use services.

Central to this vision must be a commitment to equity and social justice, and a recognition of the fundamental human rights of those who use drugs and alcohol. This will require a shift away from stigmatising and criminalising approaches to substance use, towards a public health approach that prioritises harm reduction, treatment, and support. It will also require a greater focus on the social and structural determinants of substance use, and a more integrated and comprehensive approach to addressing the needs of those who are most vulnerable and marginalised.

Ultimately, the pandemic has shown us that change is possible, and that services can adapt and innovate in the face of even the most challenging circumstances. It has also highlighted the incredible resilience, dedication, and compassion of those who work in the substance use field, and their unwavering commitment to supporting those who are most in need. As we move forward, it is important that we build on these strengths and learn from the lessons of the pandemic, to create a more equitable, responsive, and person-centred system of care for all those affected by substance use.

## Data Availability

The datasets used and/or analysed during the current study are available from the corresponding author on reasonable request.
